# Innate Lymphoid Cells in Reproductive Health and Disease

**DOI:** 10.1002/eji.70007

**Published:** 2025-07-14

**Authors:** Francesco Colucci, Marco Botta

**Affiliations:** ^1^ Department of Obstetrics and Gynaecology University of Cambridge School of Clinical Medicine, NIHR Cambridge Biomedical Research Centre, Addenbrooke's Hospital Cambridge UK; ^2^ Department of Computer Science University of Turin Turin Italy

**Keywords:** innate immunity, NK cells, placenta, reproductive immunology, trophoblast

## Abstract

Half a kilogram of immune cells reside in tissues. In the uterus, innate lymphoid cells (ILC) contribute to the cyclic destruction and repair of the mucosa. During pregnancy, uterine ILC support the formation of the placenta and the growth of the fetus. They also contribute to immune responses to pathogens. ILC respond quickly to signals of tissue perturbations and, by influencing other immune cells, they organise responses that help maintain tissue health. Their functions have been determined in the respiratory and intestinal tracts, skin, liver and adipose tissue. It is challenging to determine the function of uterine ILC because of the cyclic changes of the endometrium and the difficulties in accessing human tissues during pregnancy. We review the existing literature on the involvement of uterine ILC in physiology and pathology of the non‐pregnant endometrium as well as in pregnancy, from implantation of the fertilised egg to the tissue remodelling occurring during the first trimester and that leads to the formation of the placenta which sustains fetal growth, until parturition.

## Graphical abstract

Innate lymphoid cells contribute to the health of the uterine microenvironment, which is important for global health. Endometriosis affects 10% of women of fertile age. Millions of pregnancies have adverse outcomes every year: miscarriages, pre‐eclampsia, fetal growth restriction, preterm labour and stillbirth — all due to defective placentation. Abnormal fetal growth exposes newborns to greater risks of both acute complications and chronic diseases in adulthood. Understanding the function of uterine ILC could lead to the use of modern immunotherapies in an area of medicine where disease‐preventing and disease‐modifying treatments are virtually non‐existent.

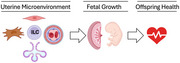

## 1 Introduction

The study of innate lymphoid cells (ILC) in the last two decades has focused our attention on the physiological role of immune cells in tissue homeostasis, besides the more studied and traditional roles in infections. ILC are found in all tissues, including the uterus, both in the endometrium and in the decidua. The endometrium is the inner lining of the uterus, shed and rebuilt at every menstrual cycle in humans, approximately 400 times between menarche and menopause. The decidua is the modified endometrium that in humans forms at the end of every cycle and, in mice, only upon implantation. For clarity, in this review, the term “uterine ILC” refers to any ILC present at any stage and in any tissue of the uterus, just like the term “uNK” refers to any type of NK cell present at any stage and in any tissue of the uterus, whereas “decidual ILC” and “decidual NK—(dNK)” refer to those ILC or NK cells present in the mucosa of the pregnant uterus. More details on the nomenclature in relation to histology, the menstrual cycle and pregnancy can be found elsewhere [1].

**FIGURE 1:**
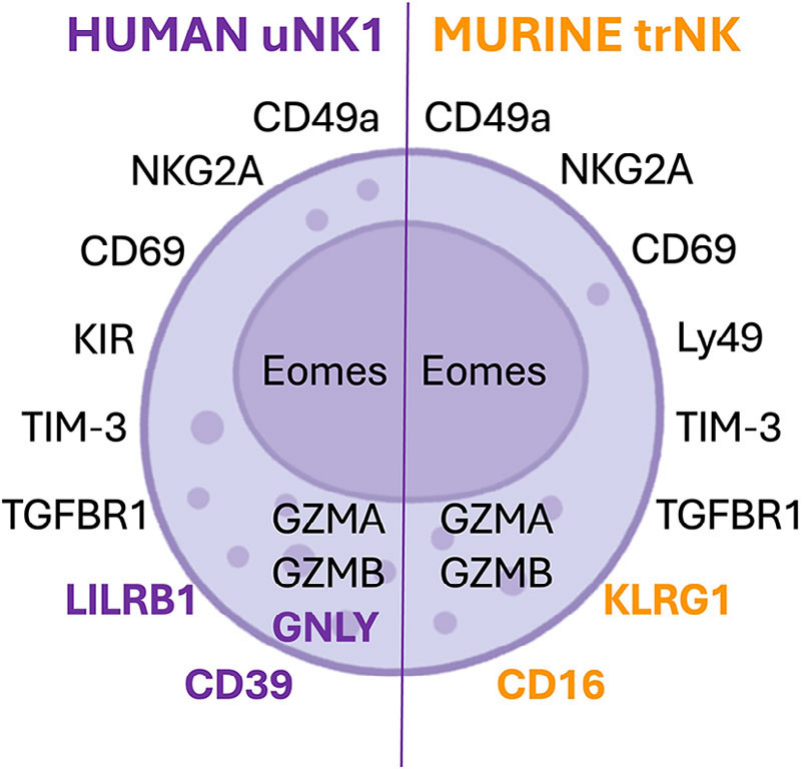
**Comparison between human uNK1 and mouse uterine trNK cells**. Shared and species‐specific markers of human uNK1 and mouse uterine trNK cells. Shared = black; Human = purple; Murine = Orange.

The wellbeing of the uterus, from menarche to menopause, is not only key for successful reproduction, but also central to women and offspring health. The number of women and children affected by conditions related to suboptimal uterine function is overwhelming. About 176 million women (10% of all women of reproductive age) suffer from endometriosis [2], a condition due to the growth of the uterine lining outside of the uterus, which causes pain and often infertility. In the UK alone, the number of women affected by endometriosis is comparable to that of women suffering from Diabetes. Every year, about 73 million miscarriages occur worldwide, affecting about 25% of all pregnancies. About 1% are recurrent miscarriages, meaning occurring at least three times in the same couple [3]. Among pregnancies that carry on successfully, about 10% are affected by poor fetal growth, with an estimated 10 million babies born too small every year. Later in life, these babies are exposed to greater risks of cardiovascular, metabolic and neuropsychiatric disorders [4]. About 4 million pregnancies are affected yearly by the hypertensive disorder preeclampsia, which causes 70,000 maternal and 500,000 infant deaths every year [5]. An estimated 13.4 million pregnancies are affected by preterm labour, which is associated with increased risk of cerebral palsy, cognitive impairments, behavioural problems, metabolic disorders, cardiovascular disease, as well as problematic feeding, respiration and growth [6]. Finally, 2 million stillbirths occur every year [7]. What is the role of ILC in the biology and pathology of the uterine microenvironment? Cells looking like immune cells were described in the uterine mucosa of animals more than 100 years ago [8]. These cells were then recognised to be of bone marrow origin more than sixty years later in humans [9] and, in 1985, identified as NK cells in mice [10]. NK cells are the founding members of the ILC family. They were discovered in the 1970s for their unique ability to spontaneously kill tumour cells [11]. However, two landmark studies in 2000 and 2006 showed that uterine NK (uNK) cells are “builders rather than killers” because, instead of spontaneously killing, they produce factors that contribute to homeostatic functions of tissue remodelling during pregnancy [12, 13]. Despite their precise functions are still to be determined, uNK cells are active regulators at the maternal–fetal interface [14]. At around the time of the first descriptions of ILC, flow cytometry data on uterine cell suspensions showed more than one type of NK cells in mice [15, 16]. Uterine ILC were formally described in 2015 in both humans and mice, including ILC1, ILC2 and ILC3 [17, 18]. uNK cells are the most abundant population in both species. In mice, there are two types of uNK cells: one essentially indistinguishable from circulating and splenic NK cells, known as conventional uNK cells (cNK), the other known as tissue‐resident uNK cells (trNK), and a third type of NK‐like ILC known as uILC1 [17]. In humans, three subsets of uNK cells can be distinguished: uNK1, uNK2 and uNK3 [19] and are defined as in Table 1. While there are differences between human and mouse uNK cells, human uNK1 and mouse trNK cells do share similarities and may well be the key cell type to successful reproduction (Figure 1).

**TABLE 1:** Markers defining subsets of human uterine NK cells.

	uNK1	uNK2	uNK3
CD49a	+	+	+
CD9	+	+	+
CD39	+	−	−
CD103	−	−	+
ITGB1	−	+	+
GZMA	High	Medium	Low
GZMB	High	Medium	Low
GNLY	High	Medium	Low
PRF1	High	Medium	Low
KIR	High	Low	Low
LILRB1	High	Low	Low
Ki67	High	Low	Low
PBX1	High	Medium	Low

Here we discuss the biology and pathology of uterine ILC, which are involved in the cyclic homeostasis of the endometrium. In pregnancy, ILC are involved in implantation and in tissue remodelling occurring in the first trimester. Tissue remodelling modifies the uterine arteries, leading to the formation of the placenta that sustains fetal growth. ILC are also thought to participate in parturition (Table 2).

**TABLE 2:** Associations of NK/ILC abnormalities with reproductive health and disease.

Condition	Mechanism	Ref.
Endometriosis	Reduced uNK activity	[23]
Endometriosis	ILC3 distant from glands and blood vessels	[25]
Endometriosis	Excess of IL‐33 and ILC2 promote fibrosis	[27, 28]
RM or Recurrent Implantation failure	Increased uNK cells	[29]
RM	Reduced frequency of Eomes+CD49a+ uNK1 (tissue‐resident) in menstrual blood	[30]
RM	Association with maternal *KIR‐A* and fetal *HLA‐C2*	[33]
Preeclampsia	Association with maternal *KIR‐A* and fetal *HLA‐C2*	[34]
Preeclampsia	Genetic association with *HLA‐B* ligand, which determines less NKG2A education	[43]
Low Birthweight	Association with maternal *KIR‐A* and fetal *HLA‐C2*	[35]
Reproductive failure	Association with reduced expression of NK receptors for trophoblast	[36]
FGR in mice	Reduced NKG2A education in uNK cells	[43]
FGR in mice	Transgenic expression of human maternal *KIR‐A* and fetal *HLA‐C2*	[46]
Spontaneous abortion in mice	Abnormal uNK activity, including reduced *Prf1*, *Gzmb*, *Gzmd*, and *Gzme* expression	[50]
Spontaneous abortion in mice	uNK cells contrast pathogenic Th17	[51]

Abbreviations: RM, recurrent miscarriage; FGR, fetal growth restriction.

## 2 Endometrial ILC

NK cells are the most abundant ILC in the endometrium, where they regulate bleeding in the non‐pregnant uterus. Endometrial stromal cells express the progesterone receptor and, in response to the hormone, they express both IL‐15 and IL‐15RA receptor, thus trans‐presenting IL‐15 to uNK cells for their expansion and differentiation [20]. Women treated with progesterone antagonists have a strong suppression of the IL‐15 pathway and a virtual absence of uNK cells [20]. All three subsets of uNK cells are present throughout the cycle, with uNK1 and uNK2 peaking at the end of the cycle, prior to implantation, and uNK3 not changing much [21, 22]. The growth of endometrium‐like tissue outside the uterus causes endometriosis, a chronic inflammatory condition that affects about 10% of all women of reproductive age and is associated with infertility, pain and increased risk of ovarian cancer. Several studies have implicated uNK cells and their receptors in endometriosis. A recent systematic review highlighted a positive correlation between endometriosis and inhibitory uNK receptors such as KIR2DL1, KIR3DL1, NKG2A, PD‐1 and their ligands, as well as a negative correlation with activating uNK cell receptors such as NKp46, NKp30 and NKG2D. This suggests that a lower uNK cell activity may be permissive for endometriosis and that uNK cell receptors may be therapeutic targets to treat endometriosis [23].

The second most abundant ILC subset in the endometrium is ILC3. ILC3 are mostly known for keeping the integrity of the intestinal mucosa, and for their ability to communicate with both the gut microbiome and food components [24]. Their role in the endometrium is unknown. Recently, ILC3 have been found to be associated with glands and blood vessels in the healthy endometrium, suggesting that they may support epithelial and endothelial cells [25]. Two subsets of CD127+ or CD127‐ ILC3, both defined as Lin‐CD56+ CD117+ CRTH2‐, were identified that change in frequency and activity according to the time of the menstrual cycle or parturition. While the CD127+ ILC3 subset is most abundant and active after menstruation, the CD127‐ ILC3 subset is more abundant and active both during menstruation and soon after parturition. This suggests that the CD127‐ ILC3 subset is involved in endometrial repair and regeneration. In patients with endometriosis, ILC3 are more distant from glands and vessels, suggesting that the lack of ILC3‐provided factors that may support epithelial and endothelial cells could be associated with the pathogenesis of endometriosis [25].

A recent high‐resolution single‐cell reference atlas of endometrial cells obtained from 63 women with and without endometriosis offers a platform to further study endometrial physiology and pathology. Integrating these data with spatial transcriptomics and large‐scale endometriosis genome‐wide association studies, the data identify decidual stromal cells and macrophages as likely dysregulated cells in endometriosis [26]

ILC2 are rare in the healthy endometrium, but they expand in both women with endometriosis and in mouse models of endometriosis. Moreover, the alarmin IL‐33, which activates ILC2 and type 2 immunity, is elevated in the serum, peritoneal fluid and endometriotic lesions of patients. Elevated IL‐33 and increased ILC2 correlate with worsening of the endometriotic lesions and fibrosis, suggesting that IL‐33 and ILC2 may be new targets for potential therapeutic intervention in endometriosis [27]. Indeed, blocking IL‐33 or its receptor is a promising non‐hormonal therapy for endometriosis, and its efficacy is currently being tested in clinical trials [27]. In a more recent study, it was shown that the IL33‐IL33receptor (ST2) axis promotes epithelial–mesenchymal transition in endometriosis via β‐catenin phosphorylation. In the same study, an allograft mouse model of endometriosis showed that IL‐33 promotes fibrosis and increases the number of lesions, whereas blocking IL‐33 or ST2 decreases the number of lesions [28].

## 3 Uterine ILC in Pregnancy

**FIGURE 2:**
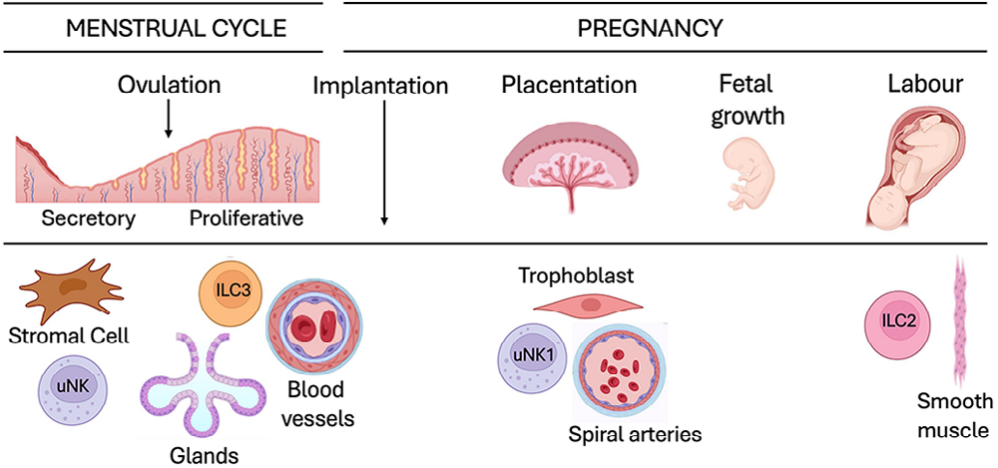
**Schematic representation of the involvement of ILC and their interactions during the menstrual cycle and in key stages of pregnancy**. During the menstrual cycle, uNK cells regulate bleeding, and ILC3 may contribute to tissue regeneration by interacting with uterine glands and blood vessels. During the first trimester of pregnancy, uNK1 interacts with trophoblast and spiral arteries, regulating trophoblast migration and differentiation as well as the formation of the placenta. The placenta itself supports fetal growth, and uNK cells may support fetal growth directly by producing growth‐promoting factors. At labour, ILC2 may interact with smooth muscle cells to promote parturition. Uterine ILC may interact with many other cell types, and these interactions are not depicted here. Created with BioRender.com.

Several articles report elevated numbers of peripheral blood NK (pbNK) or uNK cells in women with recurrent miscarriage (RM) or recurrent implantation failure (RIF), suggesting that elevated uNK activity may interfere with implantation or early pregnancy. A systematic review and meta‐analysis of the literature encompassing 60 articles concluded that the analysis of pbNK cells has very limited predictive value because the number or function of pbNK cells have no bearing on the number or function of uNK cells. The review also concluded that further research is warranted to determine the pathophysiology underlying an altered uterine microenvironment, which includes increased uNK cells in women with RM or RIF [29]. Using a non‐invasive method to measure endometrial uNK cells with the view of making that measurement a quantifiable predictor of pregnancy failure, uNK cells in the menstrual blood were phenotyped in women who had experienced RM. The frequency and phenotype of uNK cells in women with RM were compared with those in control women. The first group had significantly fewer uNK1 cells (defined in this paper as CD49a^+^Eomes^+^ tissue‐resident NK (trNK) cells) [30], confirming that RM may be due to an altered uterine microenvironment, part of which is an abnormal assortment of uNK cell subsets. These results also suggest that uNK1 may be involved in implantation. How uNK1 or other ILC subsets regulate implantation is unknown, and it is worth noting that mice lacking uNK cells or with hypofunctional uNK have normal litter sizes. Once implantation has occurred, uNK cells may support fetal growth either indirectly, through the remodelling of the spiral arteries, which lead to optimal perfusion from uterus to placenta to fetus [12, 13, 14], or directly through the production of growth‐promoting factors, including pleiotrophin and osteoglycin [31]. A schematic representation of the involvement of ILC in the menstrual cycle and key pregnancy stages is shown in Figure 2.

uNK cells but no other ILC subsets express receptors that engage with polymorphic HLA‐C ligands on invasive trophoblast cells (called extravillous trophoblast, EVT), and therefore have the potential to regulate implantation and placental formation. EVT also express non polymorphic HLA‐E and HLA‐G. T cells, of course, also may engage with polymorphic HLA ligands, however nor HLA‐A or HLA‐B are expressed by EVT. KIR and LILRB1, which bind respectively HLA‐C and HLA‐G, are mostly expressed by uNK1, whereas NKG2A, which binds HLA‐E, is expressed by all three uNK subsets [32]. Genetic epidemiology data have shown an association of certain combinations of inhibitory KIR and HLA‐C2 allotypes with adverse pregnancy outcomes, including RM [33], preeclampsia [34] and low birthweight [35]. In a study of 16 women with reproductive failure and 11 women with no reproductive problem, it was found that, during the window of implantation, uNK cells from women with reproductive failure had reduced expression of receptors that interact with HLA‐C and HLA‐G on EVT during early pregnancy. Less engagement with trophoblast ligand may cause insufficient uNK activation, which could be a feature of the suboptimal uterine microenvironment [36]. This suggests that uNK cell activation, rather than suppression, is vital for successful implantation, supporting the notion that uNK cells are active regulators at the maternal‐fetal interface [14].

Preeclampsia is a syndrome affecting pregnant women and typically manifests after the 20th week of gestation with sudden high blood pressure and signs of kidney dysfunction. It cannot be predicted, prevented or treated, unless by expelling the placenta. If untreated, it leads to eclampsia, which may end in death of mother and child. Therefore, it is an obstetrical complication because it is often accompanied by premature delivery and, sometimes, by fetal growth restriction. Women with two KIR A haplotypes and more fetal than maternal copies of HLA‐C2 allotypes have a twofold greater risk of developing pre‐eclampsia, presumably because the KIR A haplotypes, carrying seven inhibitory KIR genes, may prevent sufficient uNK cell activation upon interaction with cognate HLA‐C2 allotypes. The same genetic combinations are also more often associated with low birthweight, again suggesting that adequate uNK cell activation is key for healthy pregnancy and optimal fetal growth [37].

Determining the precise mechanisms of how uNK cell activation underpins healthy placentation is challenging due to the dynamic nature of the tissue and the difficulty to access samples. However, using trophoblast organoids, it was recently shown that EVT express receptors for and respond to uNK‐cell‐derived cytokines such as CSF1, CSF2, CCL5 and XCL1. In response to these uNK‐cell‐derived cytokines, EVT differentiate and activate genetic programmes important for placentation [38]. Therefore, genetic combinations of KIR and HLA ligands which favour appropriate activation of uNK cells may be associated with a lower risk of preeclampsia due to their favouring trophoblast differentiation and placentation.

## 4 Uterine ILC in Labour and Preterm Birth

Both innate and adaptive immune cells are involved in the physiological or pathological activation of labour, not only those cells present in the uterine mucosa and the smooth muscle layer (myometrium), but also immune cells in the cervix, the fetal membranes or in the fetus itself [39]. Preterm birth is a major determinant of neonatal morbidity and mortality, and a timely triggering of immune cells at the maternal–fetal interface is key for healthy labour. Neutrophils, macrophages and mast cells may be major mediators of the process of labour by releasing pro‐inflammatory cytokines, chemokines and matrix metalloproteinases. However, ILC may also play important roles. For their ability to induce smooth muscle contraction through secretion of type 2 cytokines, ILC2 are obvious candidates and indeed, ILC2 increase in number and percentage towards the end of pregnancy [40]. In mice, but not in humans, a physiological drop in progesterone triggers parturition. However, by administering exogenous progesterone, one can model a uterus‐intrinsic labour in mice. Using this approach, a recent study has shown that the alarmin IL‐33 drives a type 2 response, including ILC2 and eosinophils, that leads to smooth muscle contraction and labour [41]. It is tempting to speculate that this uterine response initiated by IL‐33 is similar to the one triggered by mechanical forces at work in the lungs during the first breath, when the negative pressure created by the first inhalation stimulates IL‐33 production, which induces a type 2 response [42].

## 5 Uterine ILC in Mouse Models

Mouse models of pregnancy complications are used to determine immunological mechanisms underlying pregnancy loss or other adverse pregnancy outcomes. It is important to stress that there are differences in the biology of reproduction not only between mice and humans but also among all species. The placenta is believed to have evolved multiple times, which explains the differences in anatomy and physiology among even close species. Nevertheless, both humans and mice have an invasive type of placentation, which, though much deeper in humans, is characterised by trophoblast cells invading into the maternal tissue in both humans and mice. Interestingly, tissue uNK cells are found abundantly in species with invasive placentation, where and when trophoblast invasion occurs. It is also important to notice that certain adverse pregnancy outcomes are exclusive to humans and some great apes. For example, preeclampsia does not occur in mice. However, thanks to our good understanding of comparative immunology, studying mouse immunological mechanisms in reproduction is still informative. For example, in a study of the role of NKG2A in mouse pregnancy, we found that the interaction between this inhibitory receptor and its ligand is required for optimal uNK cell education and function [43]. In pregnant mice lacking this receptor, uNK cells were hypofunctional. The uterine microenvironment was not normal, and this led to reduced blood perfusion from the uterus to the placenta to the fetuses. This was associated with a greater number of pups not reaching their full growth potential. Those pups with reduced fetal growth also showed signs of asymmetric growth restriction, meaning that the growth‐restricted fetuses were adapting to the scarce resources available by shunting the blood to the head and away from the rest of the body. While this adaptation, known as brain sparing, effectively spares the fetal brain from hypoxia, it exposes the offspring's brain to greater risks of neurodevelopmental disorders later in life [44]. Reduced blood perfusion due to incomplete arterial remodelling is a feature of human preeclampsia, and preeclampsia can also be associated with reduced fetal growth. NKG2A is a conserved receptor between humans and mice. In a large GWAS meta‐analysis of over 150,000 pregnancies, we found that women genetically programmed to use the NKG2A pathway to educate NK cells were at relatively lower risk of developing preeclampsia [43]. This suggests that uNK education through NKG2A may be a determinant of healthy pregnancy in humans too, and that some of the pathways leading to optimal uNK cell function may be conserved between humans and mice. To answer the question as to whether maternal or fetal cells educate uNK cells, we used the well‐established MHC‐deficient *β2m* KO mouse model, in which we could compare various combinations of MHC deficiency in maternal or fetal cells. We could conclude that maternal, not fetal MHC, educates uNK cells [45].

**FIGURE 3:**
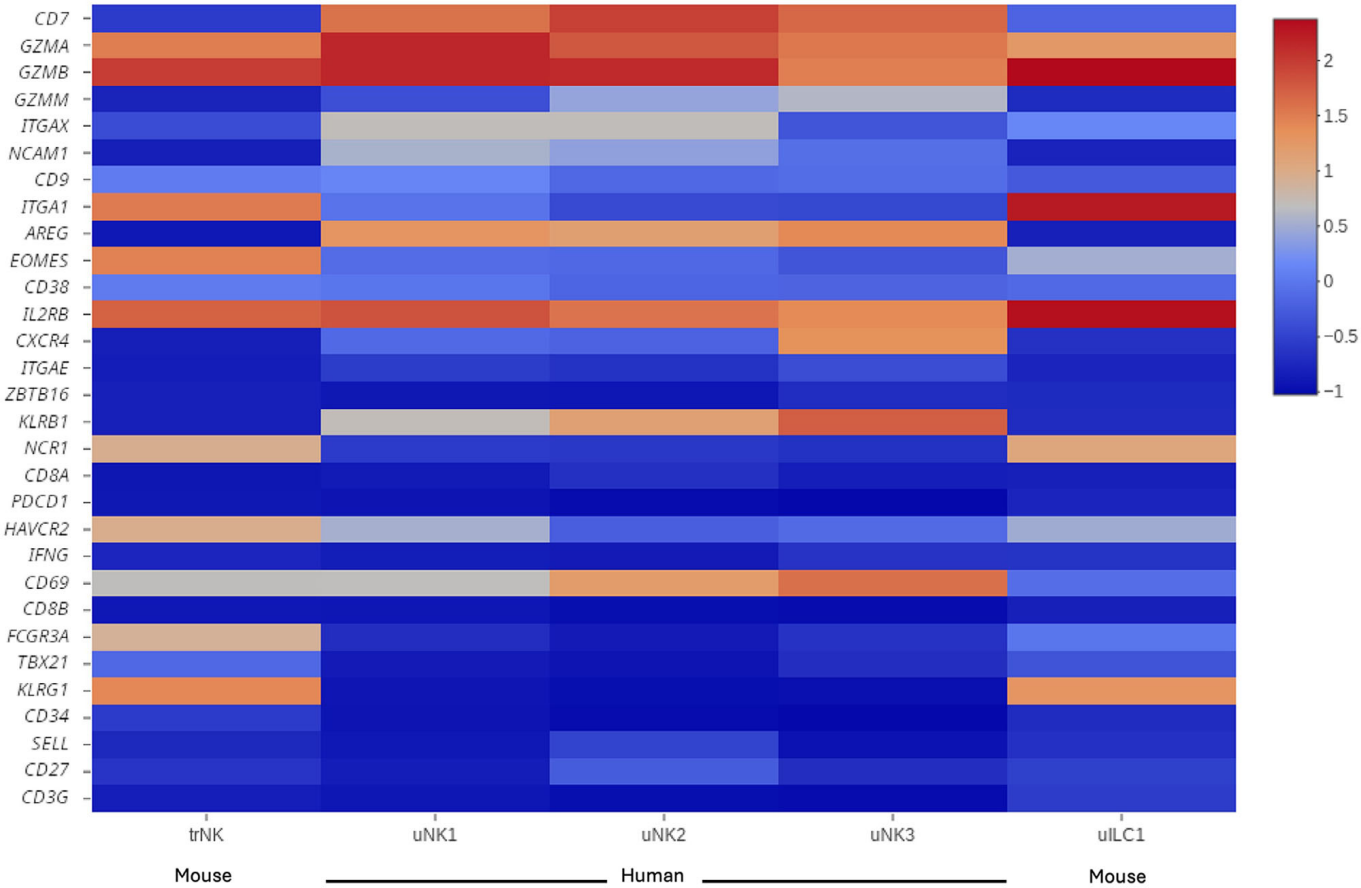
**Comparative gene expression in human and mouse uterine NK cells**. Heat map showing z‐scores of the mean log‐transformed, normalised gene expression of selected genes in uNK1‐3 of human decidua compared with mouse uterine trNK cells and uILC1. We recomputed these scores from the raw data in Ref. [47] and compared them with mouse data from Ref. [48]. For the latter dataset, we extracted gene expression values of cells positive for *Ptprc* (CD45), *Nkg7* and *Il2rb* (CD122). Within these cells, we selected trNK cells and uILC1 as in Filipovic et al. [57], using *Itga1* (CD49a), *Eomes*, and *Itga2* (CD49b) as discriminator genes (trNK express Eomes, CD49a and CD49b while uILC1 express CD49a but not CD49b or Eomes). Genes listed in this figure are present in both the human and the mouse genome, and the human nomenclature is used here for both species. From the dataset in Vento‐Tormo et al. [47], we have excluded *GNLY* because it does not exist in the murine genome. The mouse counterpart of human *CD8B is Cd8b1*, and the mouse counterpart of human *FCGR3A* is *Fcgr3*.

As indicated earlier, certain combinations of KIR and HLA‐C genes are associated with adverse pregnancy outcomes. However, mice do not express KIR or HLA genes, though they express analogous Ly49 and H‐2 genes which mediate similar functions in NK cells. To determine the underlying biology of the association between KIR and HLA‐C genes with adverse pregnancy outcome, transgenic mice were generated that express specific KIR and HLA‐C allotypes linked to preeclampsia and low birthweight [46]. Very interestingly, the interactions between these human genes expressed on maternal uNK cells (KIR2DL1) and fetal invasive trophoblast (HLA‐Cw5), in the murine microenvironment during pregnancy, recapitulated the vascular defects which may well occur in the human uterine microenvironment leading  to adverse pregnancy outcomes. Furthermore, the reduced uterine vascular adaptations during pregnancy in KIR2DL1‐HLA‐Cw5 transgenic mice had consequences on several pups that failed to reach their full potential [46]. This demonstrates that the mouse is an informative model to study the immunological mechanisms in the uterine microenvironment that lead to adverse pregnancy outcomes. Despite species differences highlighted above, the cellular composition of the uterine microenvironment has similarities in humans and mice. New technologies using unbiased approaches, such as single‐cell mass cytometry or single‐cell RNA‐sequencing, are helping to determine the landscape of the uterine microenvironment in both species, allowing comparative analysis from independent studies [47, 48] as shown in Figure 3.

The cross between CBA/j females and DBA/2 males results in significantly more fetal resorption than in control crosses between CBA/j females and BALB/c males. Fetal resorption in mice is considered akin to spontaneous abortion. The exact reasons for the high fetal resorption rate in the CBA/j x DBA/2 cross are unclear, but several observations point to immunological mechanisms [49]. A recent scRNA‐seq study comparing tissues from both crosses revealed that complement and coagulation were the most frequent GO terms, and these were also the top two pathways. Immune pathways were also among the top ones, with several immune genes downregulated in abortion tissues, including *Il1b, Ccr1* and *Ccr1/1*, but also genes expressed in ILC and uNK cells, such as *Prf1*, *Gzmb*, *Gzmd* and *Gzme* [50]. Interestingly, the immunosuppressor *Tgfβ1* was among the upregulated genes in pathological tissue [50]. With the caveat that it is hard to discern causes from consequences in pathological tissues, these data suggest that a reduced activation of immune pathways in the uterine microenvironment—including suppression of uterine ILCs—is associated with pregnancy loss. This contrasts with initial reports suggesting that immune effector cells, including NK cells, may mediate abortion in this mouse model [49]. In another study, it was suggested that NK cells prevent fetal loss in this model by contrasting the accumulation of pathogenic Th17 cells [51], thus not supporting the idea that NK cells may be directly pathogenic for the fetus. We have provided clear evidence, using the *β2m* KO model, that uNK cells do not harm the conceptus directly, even in the most favourable scenario for potential activation of NK cells, when the fetus and its trophoblast cells completely lack MHC expression, and yet retain expression of ligands for activating NK cell receptors [45].

Of course, the uterine microenvironment comprises many other immune cells. For example, regulatory T cells are thought to be crucial for maternal tolerance of the fetus in mice [52, 53], whereas it is difficult to gather such evidence in humans [54]. It is also plausible that tissue Tregs mediate non‐immunological and homeostatic functions, like they do in the skeletal muscle or the hair follicle [55]. A recent study has shown that transient Treg depletion affects vascular remodelling in mice, trophoblast invasion and uNK cell numbers, resulting in both fetal loss and growth restriction [56], suggesting an upstream role for Treg in the vascular adaptations in the pregnant uterine microenvironment.

## Conflicts of Interest

The authors declare no conflicts of interest.

## Peer Review

The peer review history for this article is available at https://publons.com/publon/10.1002/eji.70007.

## Data Availability

The data that support the findings of this study are openly available in References [47, 48] of this paper.
